# A brief review of relationship between occupational benzene exposure and hematopoietic cancer

**DOI:** 10.1186/s40557-018-0245-9

**Published:** 2018-05-10

**Authors:** Jin-Ha Yoon, Woo Seok Kwak, Yeon-Soon Ahn

**Affiliations:** 1 Department of Preventive Medicine, Yonsei University College of Medicine, Seoul, Korea; 2 The Institute for Occupational Health, Yonsei University College of Medicine, Seoul, Korea; 3 Department of Preventive Medicine, Institute of Occupational and Environmental Medicine, Yonsei University Wonju College of Medicine, 162, Ilsan-dong, Wonju, South Korea, Wonju, 26426 Korea

**Keywords:** Benzene, Hematopoietic cancer, Leukemia

## Abstract

We reviewed articles to clarify the current evidence status for 1) types of cancer which related to benzene exposure, and 2) certain benzene exposure level which might cause the hematopoietic cancers. Hematopoietic function of the bone marrow is involved in the production of all blood cells types. The benzene metabolites including benzoquinone and mucoaldehyde affect hematopoietic stem cells as well as differentiation steps of progenitor cells for each blood cell. Hence, we concluded that benzene was associated with all lymphohematic carcinogenesis. First, it is supported by biological plausibility. Second, it is supported by meta-analysis although sing study did not show relationship due to lack of sample size or statistical power. More recent studies show lesser exposed level related to risk of cancer, compare to past studies did. Actually, early studies show the risk of malignancies in workers who exposed more than 200 ppm-years. However, only 0.5 to 1 ppm-year benzene exposed show significant linking to risk of malignancies in recent study. As reviewed research articles, we concluded that the relatively lower exposure level, such as 0.5–1 ppm-year, will be considering at risk of hematopoietic cancer. However, more research needs to be done on dose-response analysis.

## Background

The International Agency for Research on Cancer (IARC) concluded that benzene exposure has sufficient carcinogenic evidence in both humans and animals study in 1987 [1]. That report shows strong evidences that benzene exposure causes acute myeloid leukemia (AML) and acute non-lymphocytic leukemia (ANLL). There are also positive association between benzene exposure and acute/chronic lymphocytic leukemia, non-Hodgkin lymphoma, and myeloma [2].

National Institute for Occupational Safety and Health (NIOSH) in USA conducted cohort study of 459 benzene exposure workers from 1940 through 1975 [3]. That study was undertaken using workers in Pliofilm manufacture, and that results supported Occupational Safety and Health Administration (OSHA) to reduce permissible exposure limit from 10 ppm to 1 ppm [4]. Exposure standard in Korea was also established at level of 10 ppm in 1986, but it was change to 1 ppm in 2003 to prevent leukemia patients from benzene exposure.

According to the Enforcement Decree of Industrial Accident Compensation Insurance Acts of specific criteria in 2003, “1 ppm or more benzene exposure during 10 years or more” are regarded as sufficient exposure level to cause occupational diseases of leukemia and multiple myeloma. Cumulative exposure exceeds 10 ppm-year is also regarded as sufficient exposure level even though the total exposure duration is below 10 years. Furthermore, if there were no record for benzene exposure in past job exposure history, the 10 years’ cumulative exposure amount is more than 1 ppm based on current job exposure concentrations is also regarded as a sufficient exposure level for occupational disease.

Recently, a Cohort study reported that even 1 ppm-year or below benzene exposed workers also suffered from leukemia [5, 6]. One ppm-year exposures are ten times lower levels of Korea stand exposure level for occupational relatedness. Hence, current Korea Act of standards for occupational diseases relatedness should be reviewed using recent studies. We reviewed articles to clarify the current evidence status for 1) types of cancer which related to benzene exposure, and 2) certain benzene exposure level which might cause the hematopoietic cancers.

## Main text

### General characteristic of benzene

Benzene is a pale yellowish liquid with molecular formula C_6_H_6_, molecular weight of 78.11 and a flammable substance with aromatic odor. It is almost insoluble in water and soluble in organic solvents and oils. Benzene reacts violently with oxidizing agent, easy to vaporize. Benzene is absorbed into the body through inhalation, skin exposure, and ingestion. In animal experiments, about 50% of aspirated benzene is absorbed into the body [7]. In the case of skin exposure, the absorption rate is low because a significant amount is vaporized before absorption, and a high uptake rate when ingested orally. Benzene is rapidly metabolized mainly in the liver and becomes water-soluble and is released into the urine within 48 h. Some of the metabolites of benzene migrate to the bone marrow. Benzene itself appears to be non-toxic, and the metabolites from the liver, especially benzoquinone and mucoaldehyde, have bone marrow toxicity [8]. These metabolites can damage DNA and produce DNA adducts. Benzene is metabolized in different concentrations. At low concentrations, much of benzene is metabolized to hydroquinone and other toxic substances than to high concentrations.

Benzene has been used as an ingredient in inks in the printing industry, in organic solvent solvents, as a starting material and intermediate for the production of rubber, lubricants, dyes, cleaners and pesticides in the chemical and pharmaceutical industries, as an additive to unleaded gasoline. A recent major use is the manufacture of organic chemicals [2]. It is mainly used for the production of styrene, phenol, cyclohexane, aniline, maleic anhydride, alkyl benzene and chlorobenzene in Europe and also for anthraquinone, hydroquinone, benzene hexachloride, benzenesulfonic acid and drugs, dyes, pesticides and plastics. It is also an intermediate of other products. In Korea, it is usually used for the production of styrene, phenol and cyclohexane. Benzene is naturally occurring in petroleum products (crude oil, gasoline, etc.) and is added to unleaded gasoline to increase the octane number of unleaded gasoline and to suppress engine knocking. Benzene content varies from country to country, but about 1–2%. Petroleum benzene content standard of Korea are currently less than 0.7% [9].

### Biological mechanism of benzene on hematopoietic cancer

Leukemia refers to the overgrowth of abnormal immature leukocytes caused by cancer derived from blood itself or bone marrow cell. Word Health Organization classified leukemia according to precursor cells of lymphocytic and myeloid [10]. Both precursor cells of lymphocyte and myeloid cells are derived from same hematopoietic stem cells by the cell differentiation process [11]. There are also scientific reports that certain cells have both characteristics of lymphoid and myeloid cell even after differentiation process [12]. Hence, we used the word of “hematopoietic cancer” for all types of blood cell malignancies.

Hematopoietic function of the bone marrow is involved in the production of all blood cells types, and hematopoietic stem cells are differentiated into the ancestral cells of each blood cell type. Thereafter, various blood cells are produced by self-renewal and differentiation of each ancestral cells. The benzene metabolites including benzoquinone and mucoaldehyde affect hematopoietic stem cells as well as differentiation steps of progenitor cells for each blood cell. Consequently, benzene metabolites may affect all leukemic stem cells of all blood cell type in every step [13, 14]. Thus, there are biological plausibility the benzene exposure and its metabolites can cause all types of hematopoietic tumors derived from hematopoietic stem cell [15].

Despite the biological causality that benzene can cause all hematopoietic cancer, IARC has reported that other blood cancers have limited relevance to benzene, except in the case of ANLL, AML [2]. The reason for this can be explained by the low incidence of diseases except ANLL.

In adults, the incidences of other malignant hematopoietic diseases except AML are too low to elucidate association from epidemiological studies. From 2006 to 2010, the incidence of AML increased rapidly after age 40, which is much higher than that of the incidence of acute lymphoblastic leukemia (ALL) [16]. Similarly, Korean’s data show high incident rate of AML compare to that of ALL [17]. Those low incidence level of ALL may explain current status of lack of epidemiological evidence between benzene exposure and ALL incidence. If the relatively low incidence is cause of lack of evidence, the large prospective cohort study will give more realistic scientific evidence. Alternatively, meta-analysis of gathering multiple studies can solve the such low incidence related problem. Hence, we estimate Meta-Standardized mortality ratio (SMR) using by studies which published IARC reports [2].

**Fig. 1:**
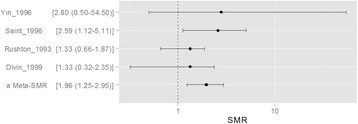
Meta-Analysis for ALL SMR using 5 studies of IARC report (ALL: acute lymphoblastic leukemia, SMR: Standardized mortality ratio)

We conducted a meta-analysis of five cohort studies in which ALL was found in more than 5 cases of exposure among 10 cohort studies in which IARC report [2]. Finally, 5 studies were selected: Yin et all 1996, Saint et all 1996, Rushton et all 1993, Divin et all 1999. Among 5 studies, only one study of Saint et all show statistical significant evidence between benzene exposure and ALL (relative risk (RR) =2.59, 95% confidence interval (CI) = 1.12 to 5.11). In contrary to Saint’s study, others did not show statistical significance. However, the pooling of those RR got statistical significance in Meta-analysis. The Meta-SMR is 1.96 and it’s 95% CI is 1.25 to 2.95 (Fig. 1). Current result of meta-analysis suggest that relatively large cohort or relatively high incidence disease can get statistical significant even those relationships did not statistical significant when using relatively small sample size or low incidence diseases.

In the same contexts, incidence of multiple myeloma is lower than that of ALL. Infante et all undertook meta-analysis using seven studies [18]. The incidences of individual studies due to benzene exposure were very low and there was no statistically significant result except for one study. However, meta-analysis showed a statistically significant and positive correlation between benzene exposure and multiple myeloma [18]. The SMR (95% CI) of each studies are 4.35 (0.1–24.2), 4.09 (1.1–10.5), 3.57 (0.4–12.9), 2.88 (0.6–8.4), 0.40 (0.1–10.7), 1.90 (0.8–3.8), 0.72 (0.2–2.1). However, the pooled estimation show significant relationship between multiple myeloma and benzene exposure (RR = 2.13, 95% CI = 1.31–3.46) [18].

In summary, the metabolism of benzene influences the whole process of hematopoietic differentiation that starts from hematopoietic stem cells. Hence, benzene exposure can cause all type of hematopoietic malignancies. Large-scale studies are required to obtain sufficient numbers of cases for other types of hematopoietic cancer, except for AML, in which epidemiological evidence is not yet sufficient. Alternatively, the results of meta-analysis result supported that biological plausibility by epidemiological concepts.

### Benzene exposure level and risk of hematopoietic malignancies

The analysis of cumulative benzene exposure and hematopoietic cancer development is as follows.

(1) Research by Wong et al. [19]

Wong et al. quantified the exposure and calculated the standardized mortality ratio (SMR) from the cohort data of workers in the factory in the US using Pliofilm (a transparent sheet made from hydrochloric rubber). Risk of AML did not be increased in 200 ppm-year exposure group, but SMRs (95% CI) for AML were sharply increased in 200 ppm-year exposure or more group. The SMRs (95% CI) for AML were 27.2 (3.3–98.0) and 98.3 (20.3–287.6) in 200–400 ppm-year and more than 400 ppm-year exposed group, respectively.

(2) Research by Hayes et al. [20]

Hayes et al. found a relative risk (RR) according to the exposure level of Acute non-lymphocytic leukemia (ANLL) in follow-up data of benzene exposed / unexposed workers at 672 plants in 12 Chinese cities. RR (95% CI) of ANLL was 1.9 (95% CI: 0.5–7.0) in less than 20 ppm-year exposed group. However, RRs (95% CI) of ANLL were 4.3 (1.1–16.0) and 3.6 (1.1–11.6) in 40–99 ppm-years and 100 ppm-year or more exposed group, respectively. The risk of ANLL was also estimated according to average level of benzene exposure. The RRs (95% CI) of ANLL were 2.0 (0.6–7.0), 5.8 (1.8–18.9) and 2.6 (0.7–9.9) in exposed group of less than 10 ppm, 10–24 ppm and more than 24 ppm, respectively. In summary, significant increment of risk for ANLL was observed in 40 ppm-year of cumulative exposure and 10 ppm of average exposure group.

(3) Study of Schnatter et al. [21]

Schnatter et al. reported a significant increase in the incidence of myelodysplastic syndrome (MDS) at low concentrations of benzene exposure after updating the cohort of Australian, Canadian, and British oil workers. The cumulative exposures of 2.93 ppm-years group show high risk of MDS compare to less than 0.35 ppm-year exposed group. The RR (95% CI) of MDS was 4.33 (1.31–14.3) in 2.93 ppm-years or more exposed group. The RR (95% CI) of MDS was 6.32 (1.32–30.2) when highest exposure level exceeded over 3 ppm.

(3) Research by Glass et al. [22, 23]

In the “Health Watch” study of Glass et al., which followed up 16,000 Australian workers from 1980 to 1998, 79 hematologic malignancies cases occurred (Glass et al., 2001). In a nested case-control study using 395 control groups, the cumulative exposure was calculated as ppm-year and divided into 5 levels to identify the risk of malignancies. The concentrations of the lowest exposed group were 0.005 ppm-year to 0.33 ppm-year, followed exposed group was 1.42 ppm-year, 3.53 ppm-year, and 7.82 ppm-year. 18 cases of malignancies were observed in the third level cumulative exposure group, and RR (95% CI) of hematologic malignancies was 2.54 (1.05–6.18). In the fourth exposed group, the RR (95% CI) did not show significant relationship, 2.20 (0.89–5.43). In contrary to that, 22 cases were observed in the fifth exposed group and RR (95% CI) was 3.32 (1.40–7.91). Glass et al. did not undertook the trend analysis. We undertook Cochran-Armitage Trend Test using reported number of cases and controls in published article of Glass et al. The *p* value of trend analysis was 0.011 (below 0.05). Hence, we concluded that cumulative exposure of 1.46 ppm-year or more significantly related to risk of hematopoietic malignancy.

Glass et al. (2005) reanalyzed this cohort and found that OR (95% CI) of workers with cumulative exposures in excess of 8 ppm-year benzene exposure was 7.2 (1.3–40.4) compare to worker who exposed less than 4 ppm-year [23]. For all types of leukemia, the risk was significantly increased in more than 2 ppm – years exposed group. In the group exposed to less than 10 years, benzene exposed group was divided by cut level of 0.5 ppm-year, 1 ppm-year, 2 ppm-year, 4 ppm-year and 8 ppm-year or more. The RR (95% CI) was 2.27 (1.14–4.54) in 0.5-1 ppm-year exposed group when comparing the risk in less than 0.5 ppm-year exposed group. Thus, workers who exposed benzene only 0.5 ppm-year or more during 10 years are at risk of ANLL. In other words, exposed to 0.05 ppm for 10 years or more can be considered to be associated with a higher risk of hematopoietic cancers in that study.

In summary, more recent studies show lesser exposed level related to risk of cancer, compare to past studies did. Actually, early studies show the risk of malignancies in workers who exposed more than 200 ppm-years. However, only 0.5 to 1 ppm-year benzene exposed show significant linking to risk of malignancies in recent study. This phenomenon may be observed in other occupational diseases such as heavy metal induced diseases. In the high exposure period, it is not easy to find non-exposure group. Hence, the non-healthy reference group might be control group in relatively high exposed period or society. Non-healthy reference or exposed reference group may make underestimate of risk. Hence, the cut level of statistical significant easy upward in that period. In the contrary to that, the lower exposed period can find heathy reference and non-exposed reference in same factory or same research design. It supported by research of Glass et al. Hence, the recently result that 0.5–1 ppm-year benzene exposure significantly related to hematopoietic cancer can give us important message. Hence, recent studies suggest that 0.5 to 1 ppm-year benzene exposure significantly related to risk of lymphohematic malignancies.

## Conclusions

Finally, it was concluded that benzene was associated with all lymphohematic carcinogenesis. First, it is supported by biological plausibility. Second, it is supported by meta-analysis although sing study did not show relationship due to lack of sample size or power. In relation between the cumulative exposure of benzene and hematopoietic cancer, the relatively lower exposure level, such as 0.5–1 ppm-year, will be considering at risk level. However, more research needs to be done on dose-response analysis.

## Abbreviations

ALL: acute lymphoblastic leukemia
AML: acute myeloid leukemia
ANLL: acute non-lymphocytic leukemia
CI: confidence interval
IARC: International Agency for Research on Cancer
NIOSH: National Institute for Occupational Safety and Health
OSHA: Occupational Safety and Health Administration
RR: Relative risk
SMR: Standardized mortality ratio
